# Mid- and far-infrared data for the analysis of Australian plant exudates

**DOI:** 10.1016/j.dib.2025.111830

**Published:** 2025-06-24

**Authors:** Abigail K. Mann, Dominique Appadoo, Claire E. Lenehan, Rachel S. Popelka-Filcoff

**Affiliations:** a Institute for Nanoscale Science and Technology, College of Science and Engineering, Flinders University, Bedford Park, South Australia 5042, Australia; b College of Science and Engineering, Flinders University, Bedford Park, South Australia 5042, Australia; c Australian Synchrotron, Clayton, Victoria 3168, Australia; d School of Geography, Earth and Atmospheric Sciences, University of Melbourne, Melbourne, Victoria, Australia 3010

**Keywords:** Plant exudates, Australian archaeology, Synchrotron THz spectroscopy, Infrared spectroscopy

## Abstract

Plant exudates have been used around the world for cultural expression and various applications throughout the archaeological record and continue today. Indigenous Australians utilize specific plant exudates for their physiochemical properties and as a fundamental connection to Country. This manuscript contains data related to the analysis of aged Australian native plant exudates, using an assemblage from turn of the 20^th^ century with provenance information but no further information on the collectors. Data from these aged samples are augmented by parallel examples from worldwide locations that have been more extensively characterized. Data were acquired via laboratory-based mid-infrared spectroscopy (mid-IR) and synchrotron-based far-infrared spectroscopy (far-IR). Spectral data are presented, organised by genera with multiple samples (*Xanthorrhoea, Aracauria, Acacia, Callitris, Eucalyptus*) for both mid- and far-IR regions to allow direct comparisons of the fingerprint areas for both spectral regions. All spectra were normalised to their highest and lowest values for presentation. Further comparisons can be made with future work on native Australian plant exudates in collections and cultural heritage materials, to identify their genera and species. This manuscript presents the collected spectral data in the mid and far infrared.

Specifications Table

**Table:** 

Subject	Earth & Environmental Sciences
Specific subject area	Archaeological science, Infrared Spectroscopy, Australian archaeology.
Type of data	Data (.xls), Tables (.docx), Figures (.tiff) Raw data from instrument, Processed data.
Data collection	Mid-IR: Powders were analyzed using a ThermoFisher Scientific Nicolet Nexus 870 FT-IR instrument with a Thermo Smart Orbit™ diamond ATR attachment. Spectra were acquired 3800-600 cm^−1^ at a 1 cm^−1^ resolution. Preprocessing included ATR (RI 2.418), baseline correction and normalization (1,0). Far IR: Spectra were acquired between 1000 - 40 cm^−1^, with a 4.0 cm^−1^ resolution on pressed powdered resin using a Bruker IFS 125/HR Fourier Transform (FT) spectrometer (Australian Synchrotron THz beamline) with Pike Technologies diamond ATR sampling accessory (6µm) (RI 2.4). Preprocessing included averaging 1000 spectra, background subtraction, baseline correction and normalization (1,0). Spectra were cropped between 700-50 cm^−1^ reflecting the optimal range for the detector used.
Data source location	Melbourne, Australia.
Data accessibility	Repository name: University of Melbourne Figshare Data identification number:10.26188/26878414 Direct URL to data: http://doi.org/10.26188/26878414
Related research article	Mann, A. K.; Appadoo, D.; Lenehan, C. E.; Popelka-Filcoff, R. S. Combining ATR far- and mid-infrared spectroscopy to distinguish native Australian plant exudates for cultural heritage analysis. Journal of Archaeological Science 2025, 176, 106167. DOI: https://doi.org/10.1016/j.jas.2025.106167.

## 1 Value of the Data

• These data are valuable contribution to the literature as they are the first comprehensive IR analysis of a unique collection of aged (>100-year-old) plant exudate samples from Australia. Analysis of this collection provides insights into plant exudates which are endemic to Australia and already have undergone aging for several decades.
• This data set is also the first analysis of plant exudate materials by far-IR analysis techniques, adding value through the development of new analytical approaches to cultural materials. This far-IR method can have additional applications for related materials in Australia and worldwide.
• This dataset adds to worldwide data on plant exudate composition, contributing to valuable analytical and traditional knowledge on plant exudate chemistry. The dataset allows comparisons with extant data sets from other global locations including comparisons with related species in other continents, and Australian species in archaeological contexts, community, museum and cultural heritage collections.
• Researchers worldwide can make further comparisons to global plant exudate data sets to better understand parallel chemistries arising from different or related genera or species worldwide.

## 2 Background

Plant exudates are a key material in worldwide cultures for cultural and practical uses, where the exudate chemistry often directs their uses [1]. Indigenous Australians have used and continue to utilize plant exudates in a variety of functions, and many instances are found in the archaeological record. Extensive analytical work has to characterized plant exudates from Europe, and Asia; however, Australian native plant exudates are significantly understudied as compared to parallel materials from other parts of the world, by any type of analytical characterisation, with the exception of a small number of studies e.g. [[1], [2], [3]]. This study focused on the comprehensive characterisation of mid-and far-IR analysis of a historic collection of aged plant exudates to identify their key functional group components [1,4]. Information from both IR regions provides complementary information on functional groups and changes in vibrational state for a more comprehensive characterization of these complex cultural materials and enhanced understanding of their cultural uses related to their chemistry*.*

## 3 Data Description

This article describes the full dataset of the linked data set of spectral data and statistical analyses of worldwide and Australian plant exudates. Commercial samples and native Australian plant exudate materials were analyzed in the same way using mid-IR and far-IR. For more details see Mann et al. [4]. The original and processed data can be found on Figshare [5].

### 3.1 Analysis of Australian native plant exudates

A table of the historical collection organized by genus and species is summarized in Table 1. The full details of collection are detailed in [1,4].

**Table 1:** Table of Australian exudates from the historical collection analyzed in this study. Genus and species of materials are reported as received.

Genus	Species
***Acacia***	*Bakeri, Excelsa* (x2), *Longifolia, Podalyriifolia*
***Aracauria***	*Bidwilli, Cunninghamii, Sp*
***Callitris***	*Alcatris, Glauca, Robusta, Sanesae, Verrucosa*
***Eucalyptus***	*Bridgesiana, Albens, Amygdalina, Calophylla, Faludosa, Largiflorens, Linearis, Melliodora, Morrisii, oleosa, Populifolia, Punctata, Radiata, Smithii, Tereticornis, Trachyphloia, Vardata*
***Xanthorrhoea***	*Arborea* (x2), *Australia*
***Canarium***	*Australasicum*
***Erythrophleum***	*Labonchorii*
***Grevillea***	*Striata*
***Myoporium***	*Viscosum*
***Sterculia***	*Diversifolia*
***Eucalyptus (Kino)***	*Corymbosa, Dealbata, Delegatensis, Hemiphloria, Oreades, Phlebophylla, Siderophloia, Sideroxylon, Woollsiana, Hybrid (Woollsiana/Microcarpa)*

### 3.2 Mid-IR spectra by genera

The following figures are plots of the mid-IR spectra organised by genera to allow observation of key spectral features that are unique to each genus. There are multiple figures for the *Eucalyptus* genera.

### 3.3 Far-IR spectra by genera

The following figures are plots of the far-IR spectra organised by genera to allow observation of key spectral features that are unique to each genus. There are multiple figures for the *Eucalyptus* genera.

## 4 Experimental Design, Materials and Methods

### 4.1 Experimental

#### 4.1.1 Mid IR data collection of plant exudate (Lab based IR)

Infrared (IR) spectra were acquired in the range of 4000-525cm^−1^ with a resolution of 1 cm^−1^ by placing approximately 0.1g of finely ground resin samples onto a Thermo Smart Orbit™ (Bayly St, Mulwala, NSW) diamond ATR attachment. This attachment was coupled to a ThermoFisher Scientific Nicolet Nexus 870 FT-IR (Fourier transform infrared) instrument (Waltham, Massachusetts, United States) with Nicolet OMNIC software interface. The analysis was undertaken using a deuterated triglycine sulfate (DTGS) detector with a KBr beam splitter. The ATR attachment was used as a non-destructive alternative to the conventional transmission FTIR spectroscopy. Each spectra presented is an average spectrum generated from the accumulation of 128 individual spectra, with a subtracted background average spectrum from 128 individual spectra.

#### 4.1.2 Far-IR data collection of plant exudates (Synchrotron analysis)

Circular disks (13 mm diameter) were formed from approximately 0.1 g of powdered resin, without the addition of a binder, by pressing under a pressure of 10 tonnes using a standard die set and a PIKE Auto-crusher pellet press (Madison, WI, USA).The generated disk was positioned on a Pike Technologies Diamond ATR sampling accessory in combination with a Bruker IFS 125/HR Fourier Transform (FT) spectrometer at the Australian Synchrotron THz Far-Infrared beamline. The infrared source was generated by synchrotron radiation, operating at a beam current of 200.1 mA and a scanner velocity of 80.0 kHz, at a controlled temperature of 24.8°C. The infrared beam was directed at an angle of 45 degrees, with a refractive index measured at 2.4. Far-infrared spectra was collected over a range of 1000 cm⁻¹ to 40 cm⁻¹, maintaining a resolution of 4.0 cm⁻¹ with a total of 1000 scans being averaged and subtracted from an average background (1000 scans) to generate the raw spectrum for each exudate sample. The data was cropped to show between 700 cm^−1^ and 50 cm^−1^ to account for detector limits.

#### 4.1.3 Data visualization and absorbance peak identification

Absorbance data was plotted against wavenumber for visual inspection, interpretation, and assessment for further processing of the spectra. The data underwent this assessment before being subjected to pre-processing techniques using Bruker OPUS version 8.2.28 [[6], [7], [8]].

#### 4.1.4 Mid-IR data pre-processing

Mid-infrared (mid-IR) spectra acquired for each resin sample was subjected to several pre-processing steps before analysis, which allowed for effective comparison of spectra across all species. The efficacy of pre-processing techniques and validation that the absorbance peaks corresponded accurately to the original spectra was visually monitored. Pre-processing techniques included an ATR correction using the refractive index (RI) of 2.418 (diamond) which was then followed by a baseline correction, reducing, and compensating for sloping effects which occurred in the data. Corrected spectra then underwent normalization [1,0] to obtain consistent comparison for relative peak intensity and position in addition to ensuring all variables contributed equally to further statistical analysis. Visual presentation of the spectra was achieved through Origin Pro 9 version 9E, where an offset was applied between spectra for clarity and comparison of spectral features within the genera.

#### 4.1.5 Mid-IR and Far-IR data analysis

Preprocessing of the far infrared (Far IR) spectra included normalization of the absorbance spectra to a range between 0 and 1. The region of the spectra used for further analysis was between 700 cm⁻¹ and 50 cm⁻¹, with data outside of these regions removed as it was outside the detector capabilities, which was observed as the background approaching 0 intensity outside of this region. A baseline correction was also applied, which was visually monitored for suitability.

## Limitations

As with many natural history and cultural collections, materials for the sample sets are limited. This particular aged plant collection is wide-ranging, and most samples have several grams of material. However, it is not possible to replicate or generate more of this original sample set due to its age and limited data about its original collection over a hundred years ago (Fig. 1, Fig. 2, Fig. 3, Fig. 4, Fig. 5, Fig. 6, Fig. 7, Fig. 8, Fig. 9, Fig. 10, Fig. 11, Fig. 12, Fig. 13, Fig. 14, Fig. 15, Fig. 16, Fig. 17, Fig. 18.).

**Fig. 1:**
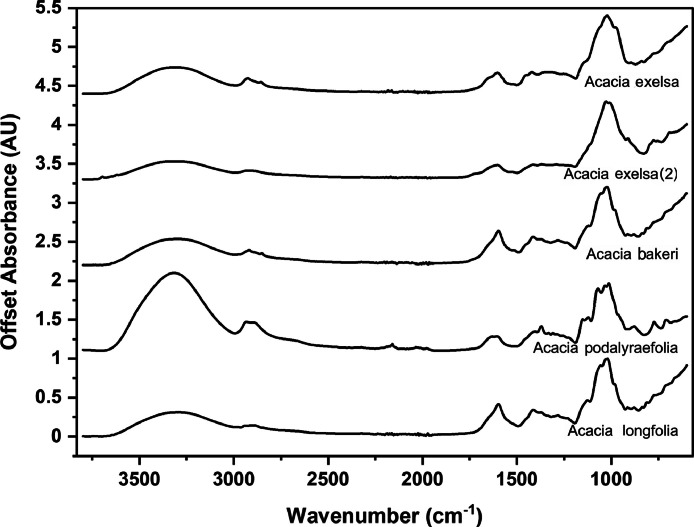
Mid-IR spectra for *Acacia* genera.

**Fig. 2:**
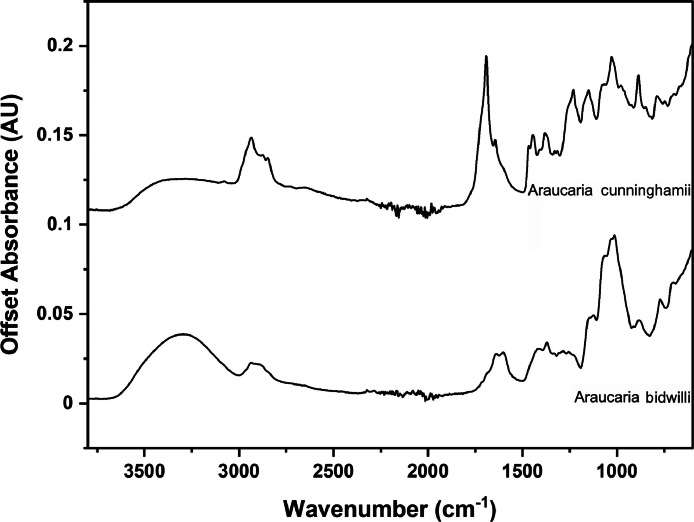
Mid-IR spectra for *Aracauria* genera.

**Fig. 3:**
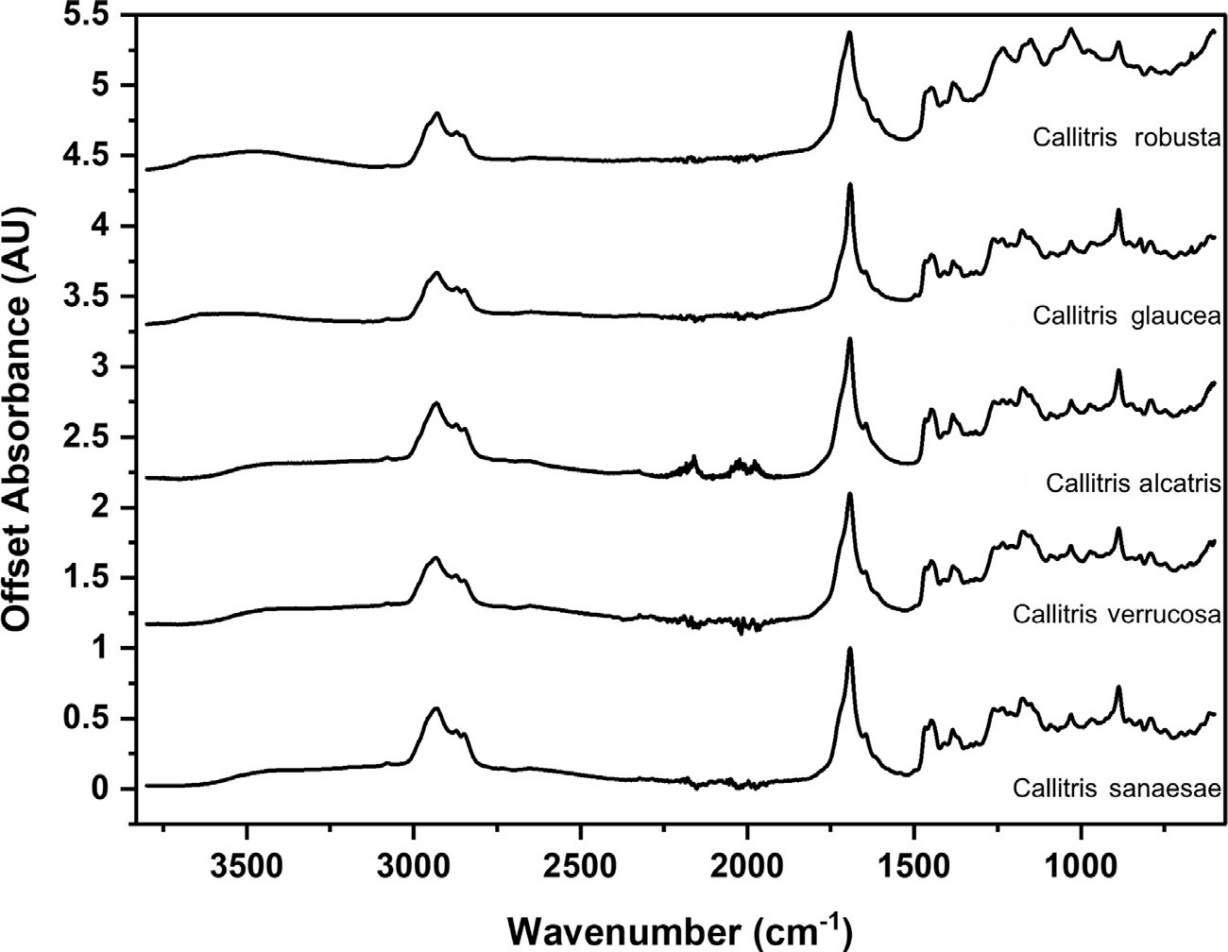
Mid-IR spectra for *Callitris* genera.

**Fig. 4:**
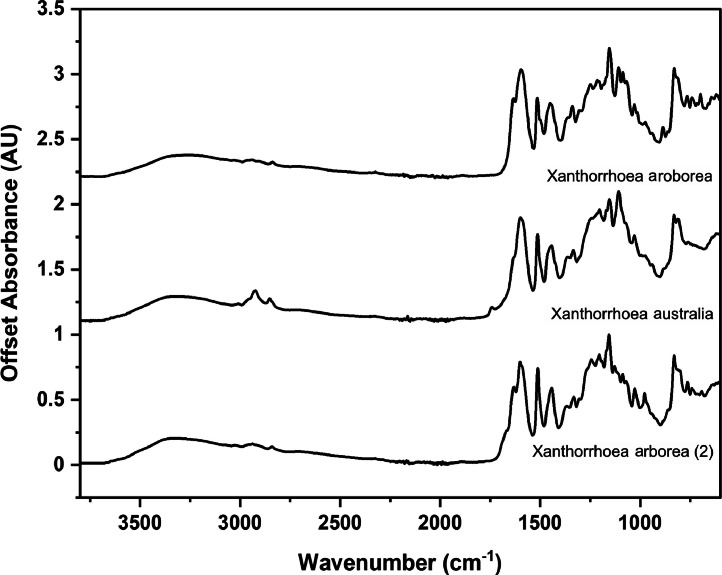
Mid-IR spectra for *Xanthorrhoea* genera.

**Fig. 5:**
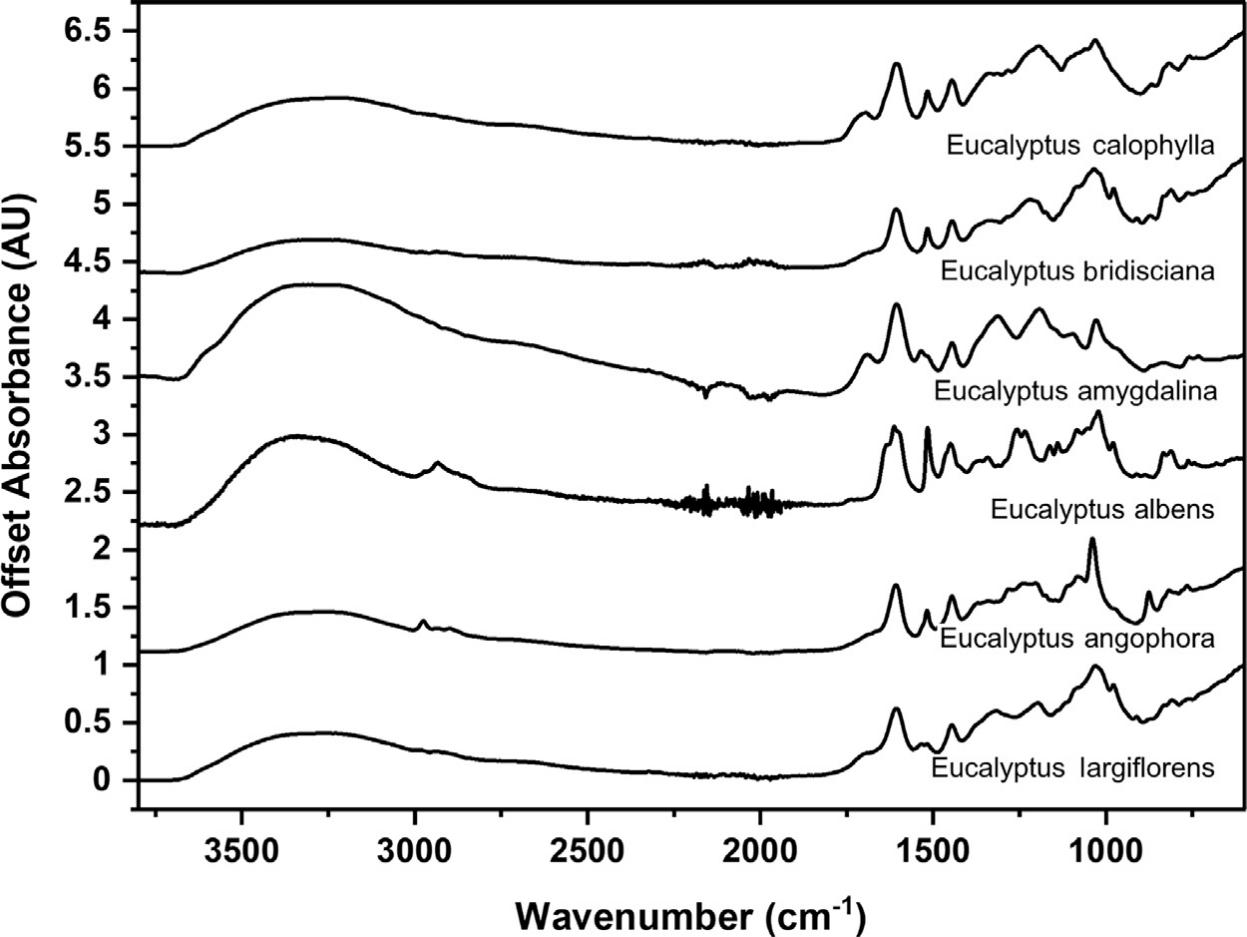
Mid-IR spectra for *Eucalyptus* genera (1).

**Fig. 6:**
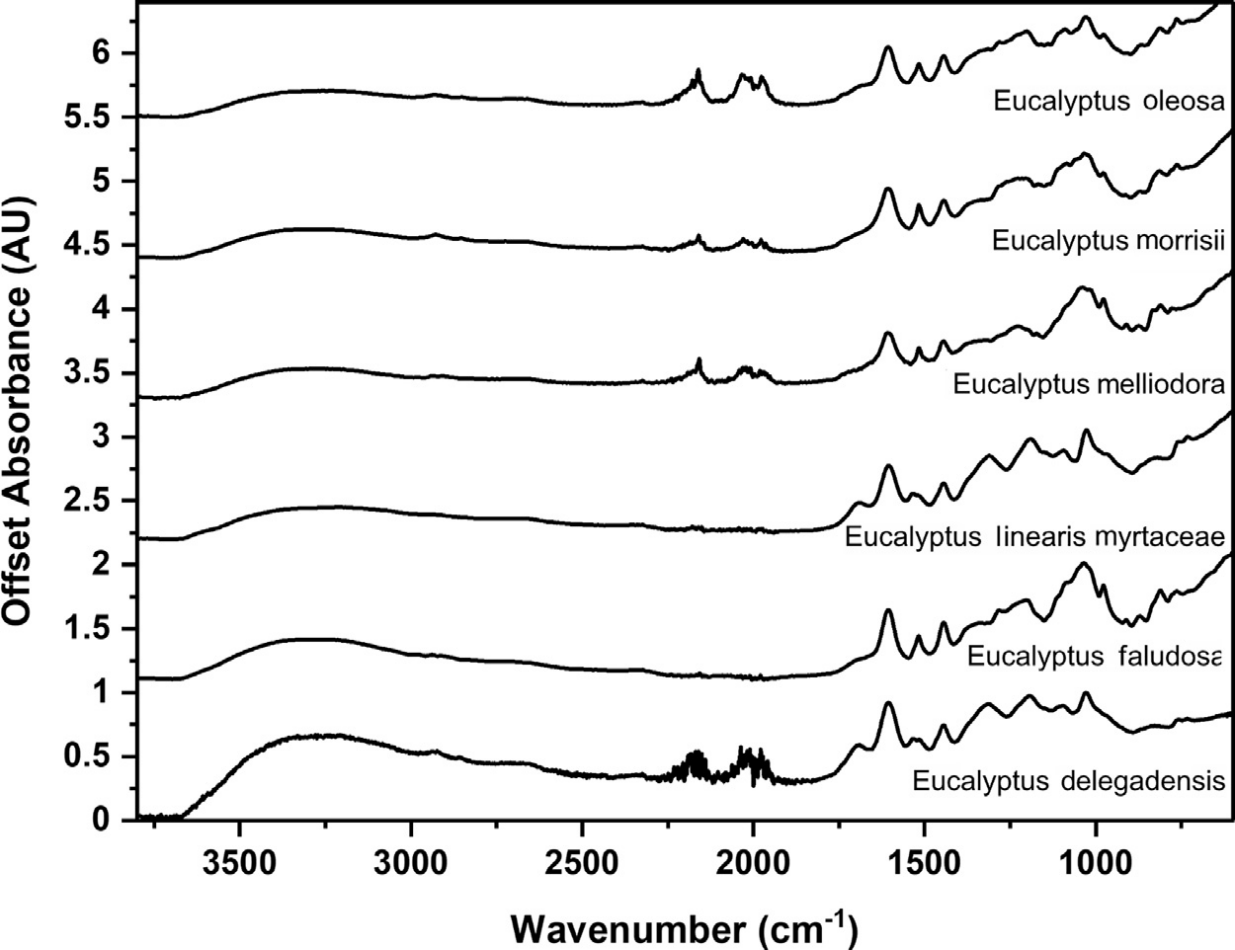
Mid-IR spectra for *Eucalyptus* genera (2).

**Fig. 7:**
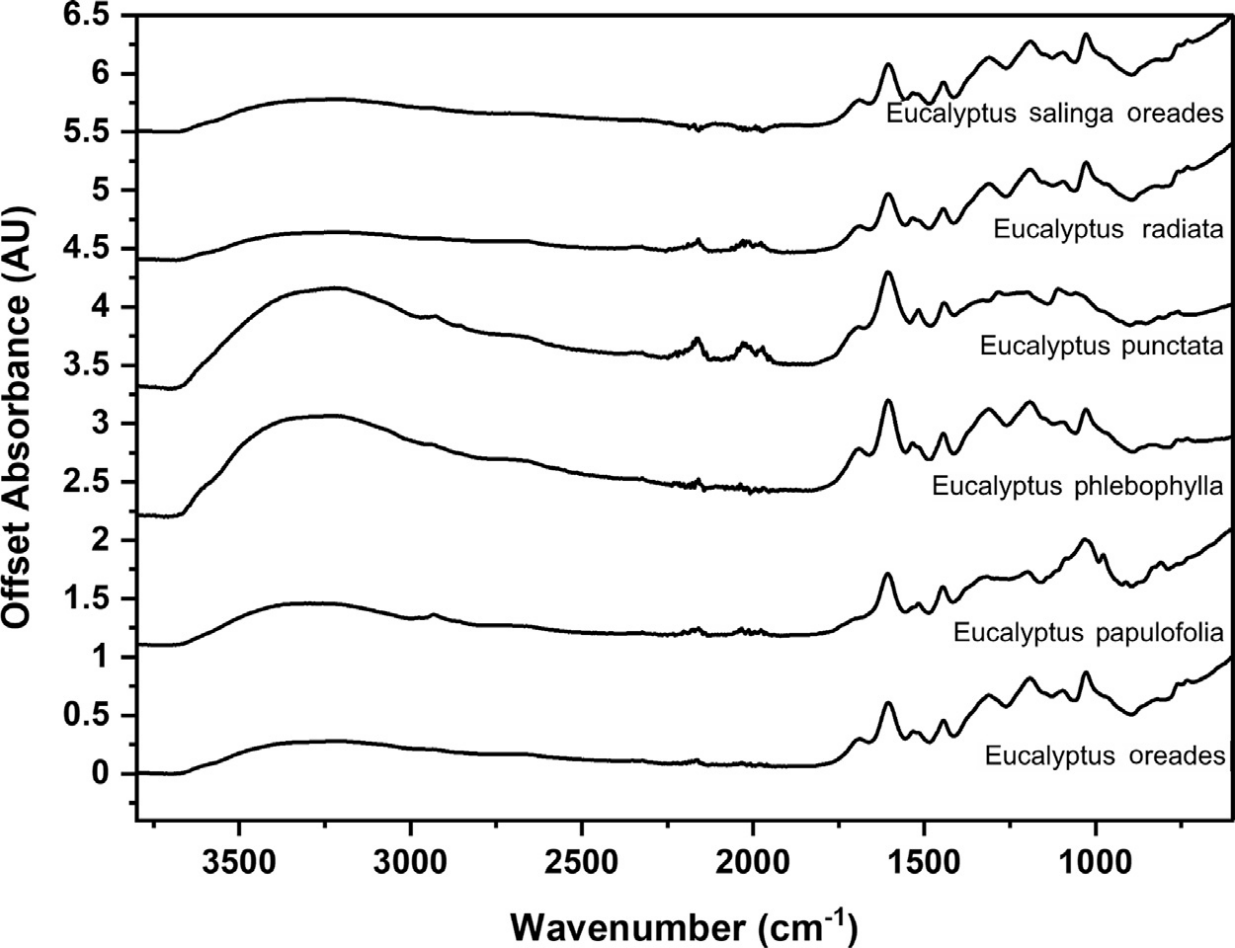
Mid-IR spectra for *Eucalyptus* genera (3).

**Fig. 8:**
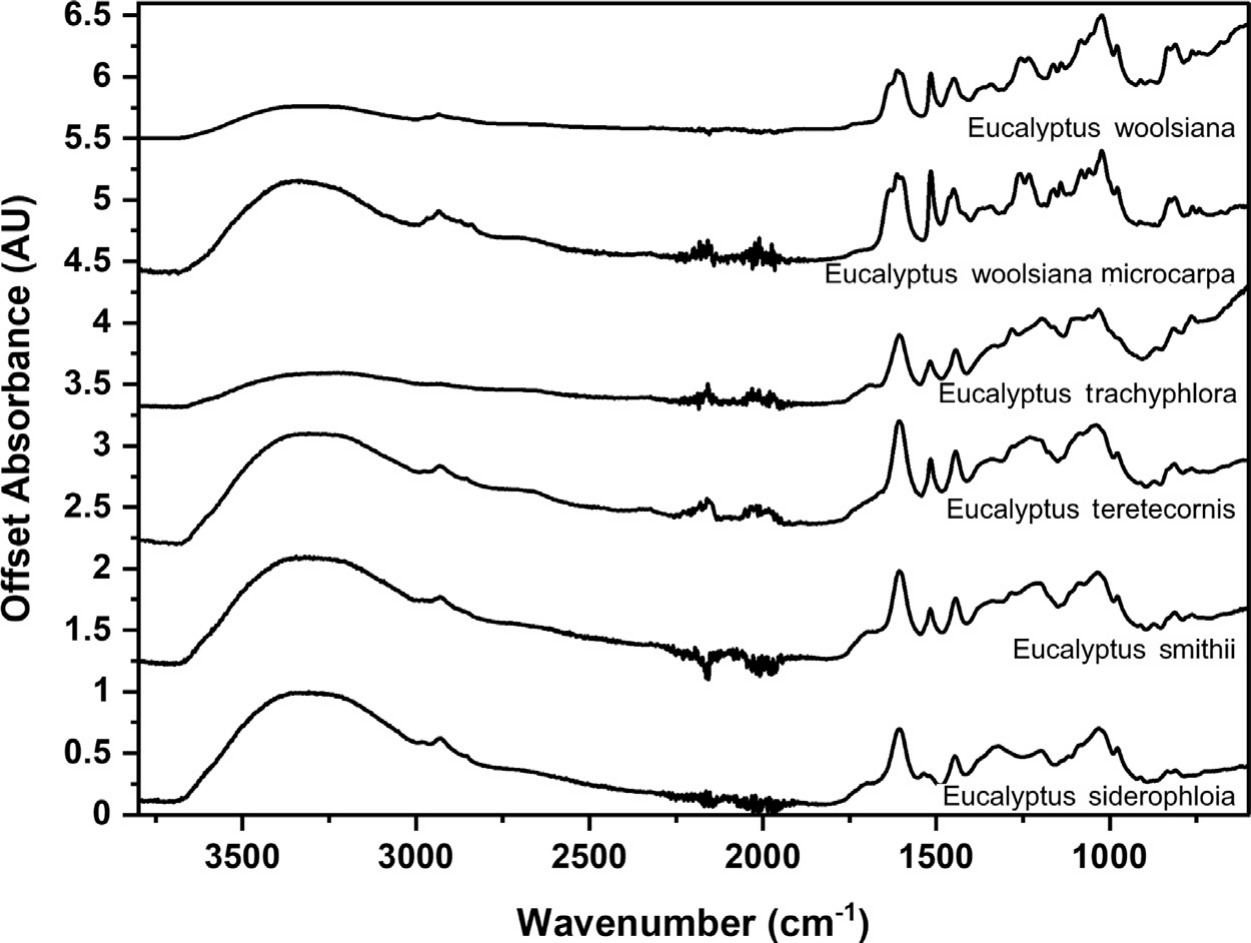
Mid-IR spectra for *Eucalyptus* genera (4).

**Fig. 9:**
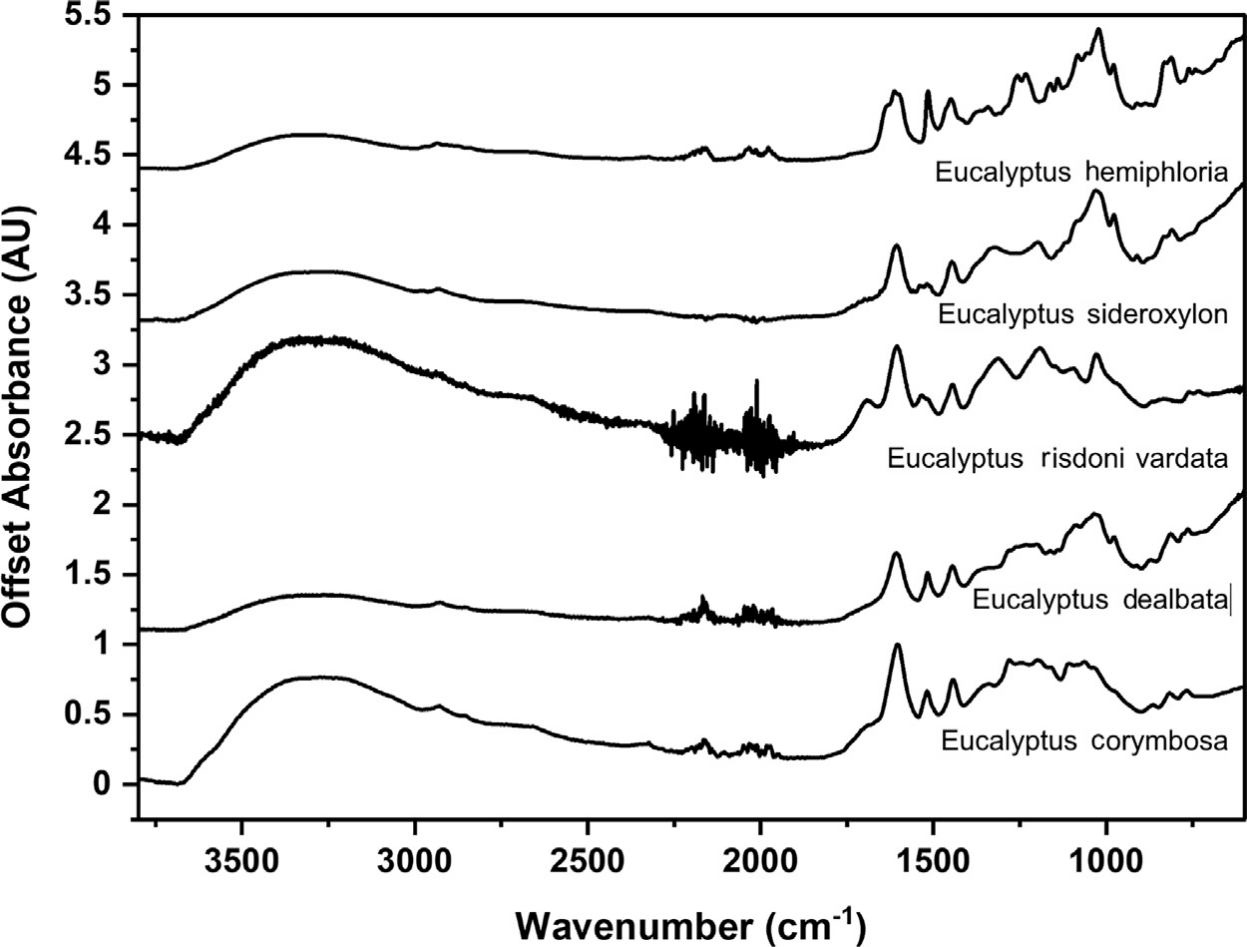
Mid-IR spectra for *Eucalyptus* genera (5).

**Fig. 10:**
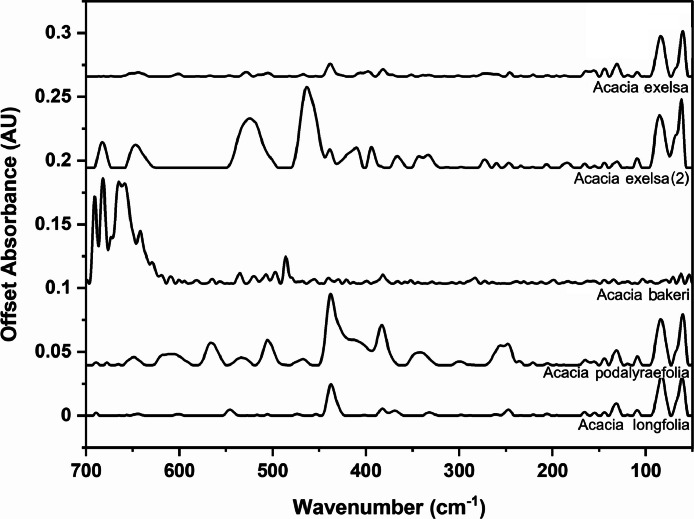
Far-IR spectra for *Acacia* genera.

**Fig. 11:**
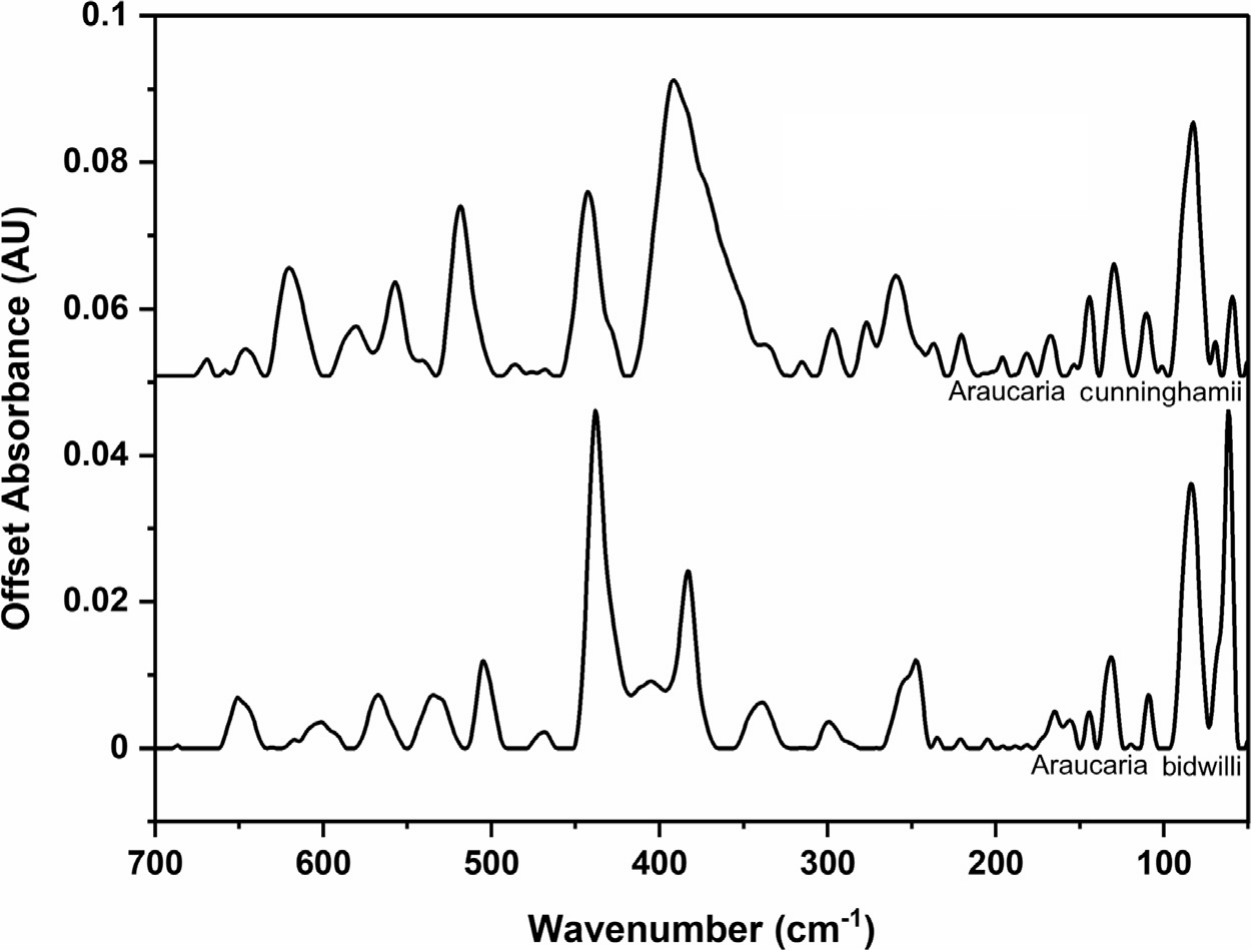
Far-IR spectra for *Aracauria* genera.

**Fig. 12:**
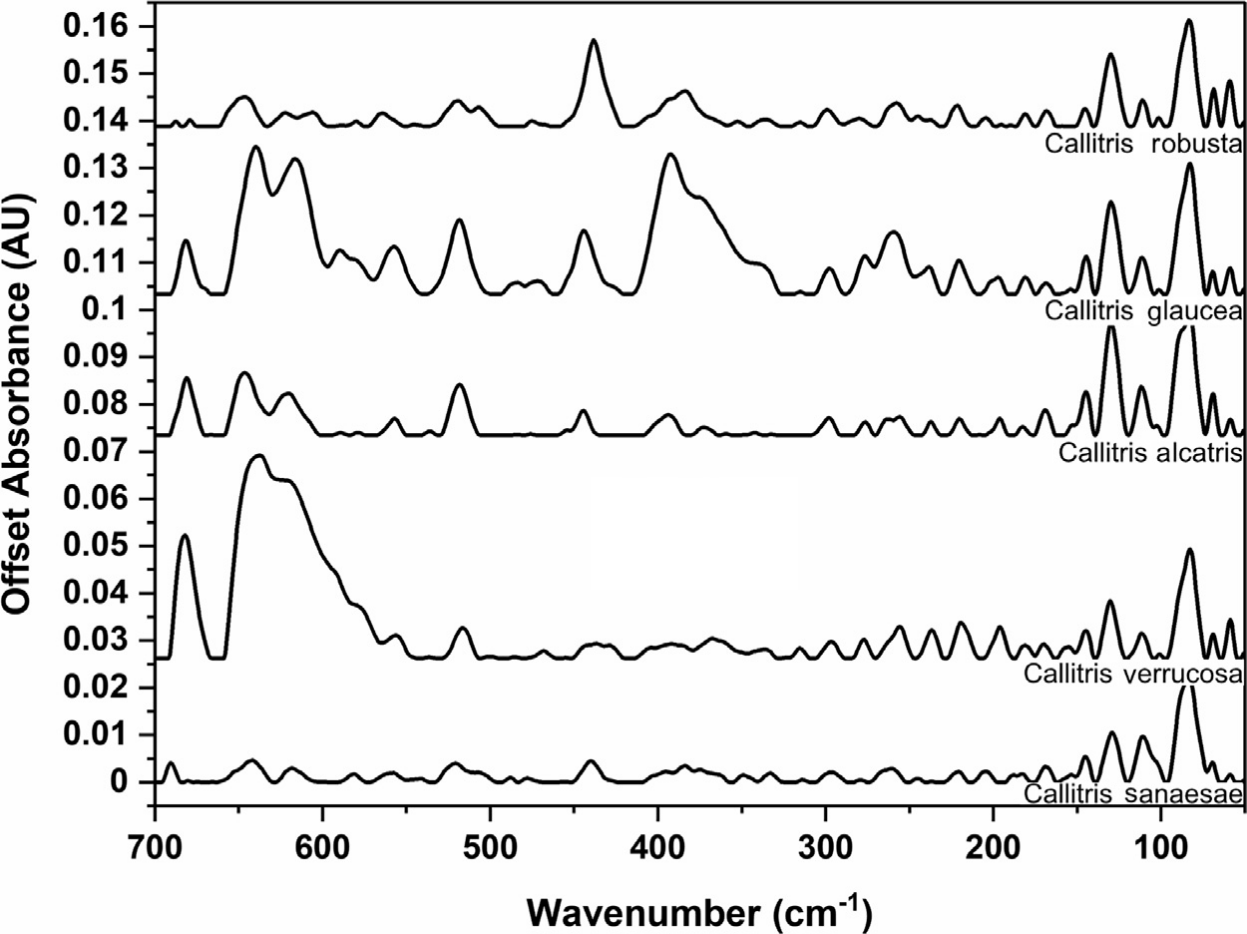
Far-IR spectra for *Callitris* genera.

**Fig. 13:**
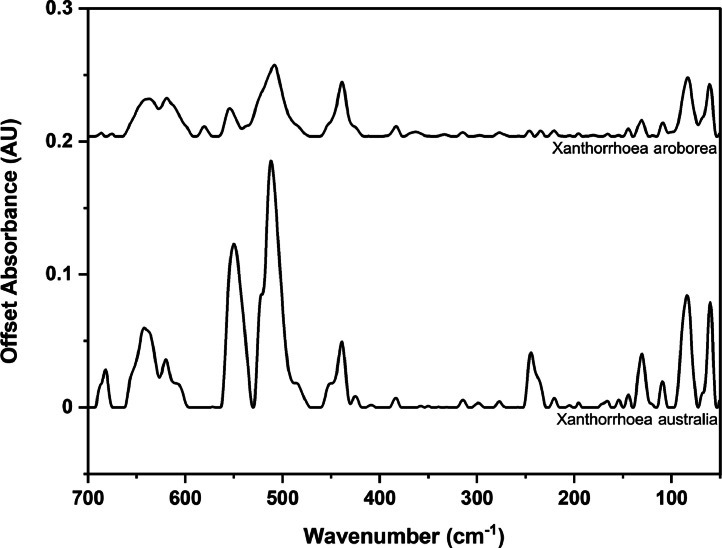
Far-IR spectra for *Xanthorrhoea* genera.

**Fig. 14:**
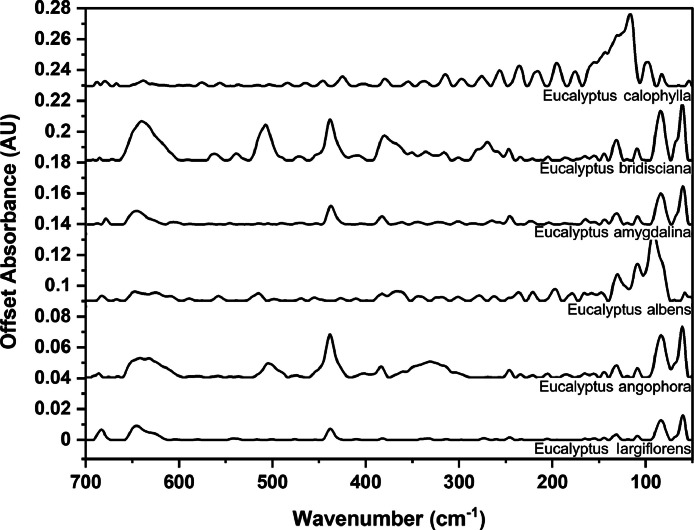
Far-IR spectra for *Eucalyptus* genera (1).

**Fig. 15:**
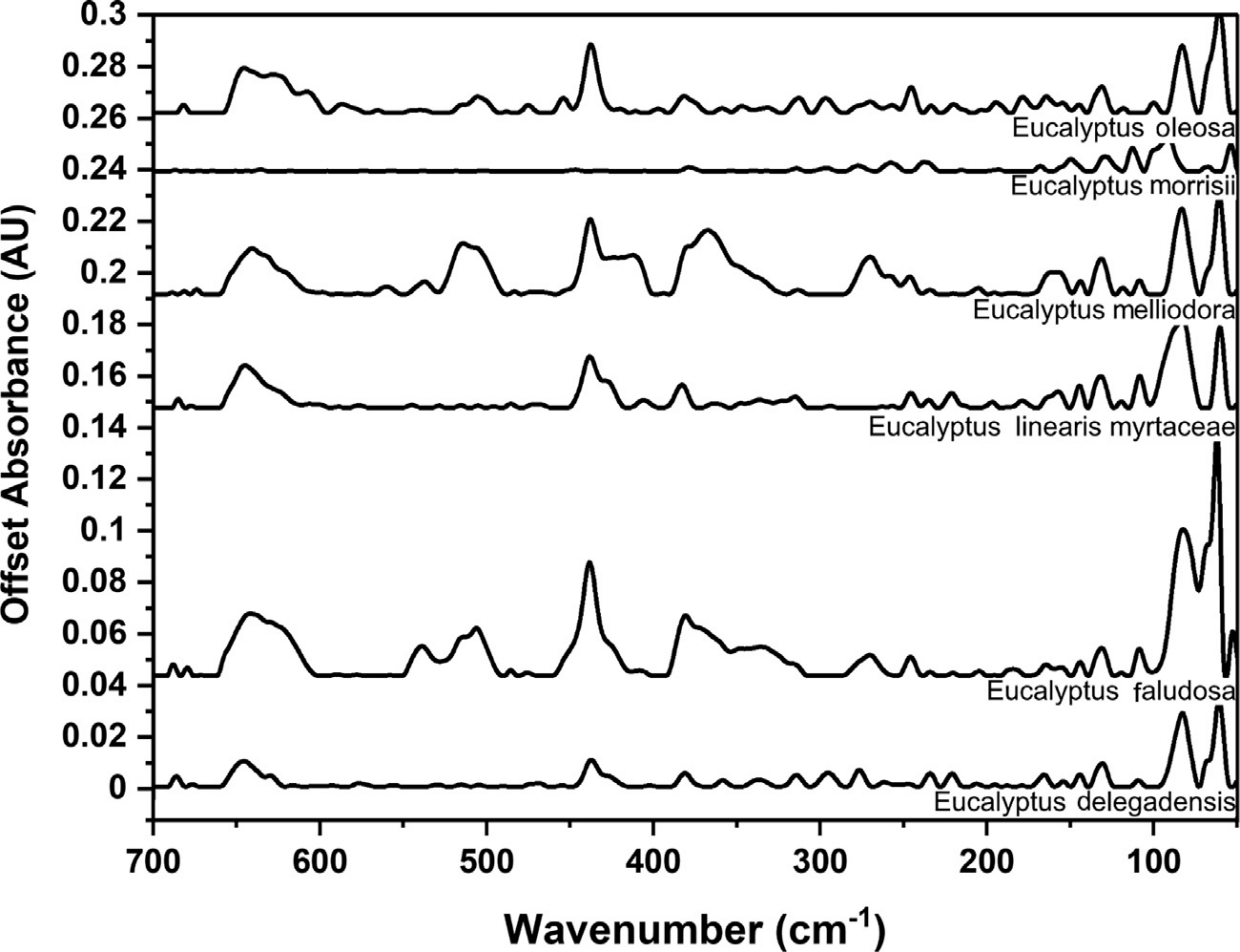
Far-IR spectra for *Eucalyptus* genera (2).

**Fig. 16:**
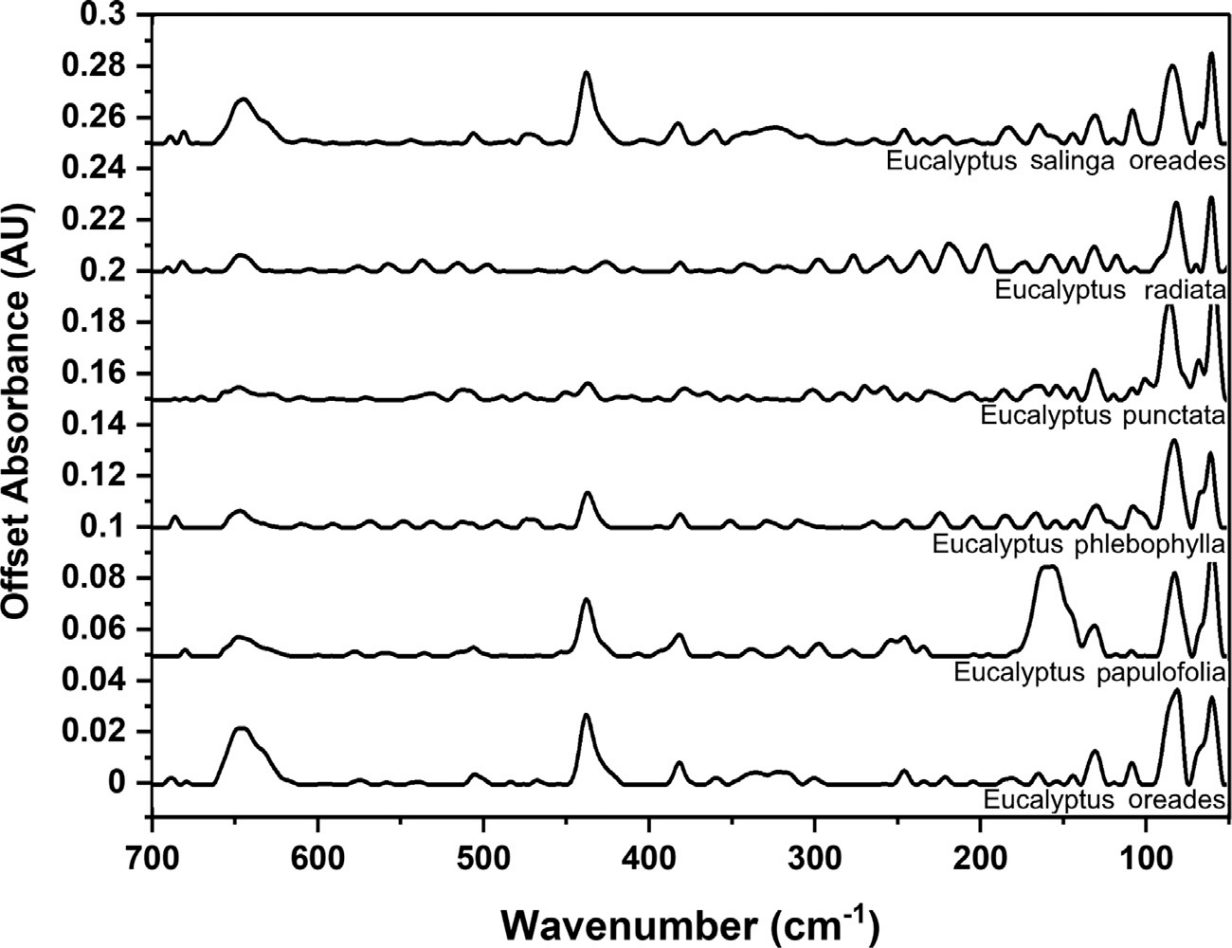
Far-IR spectra for *Eucalyptus* genera (3).

**Fig. 17:**
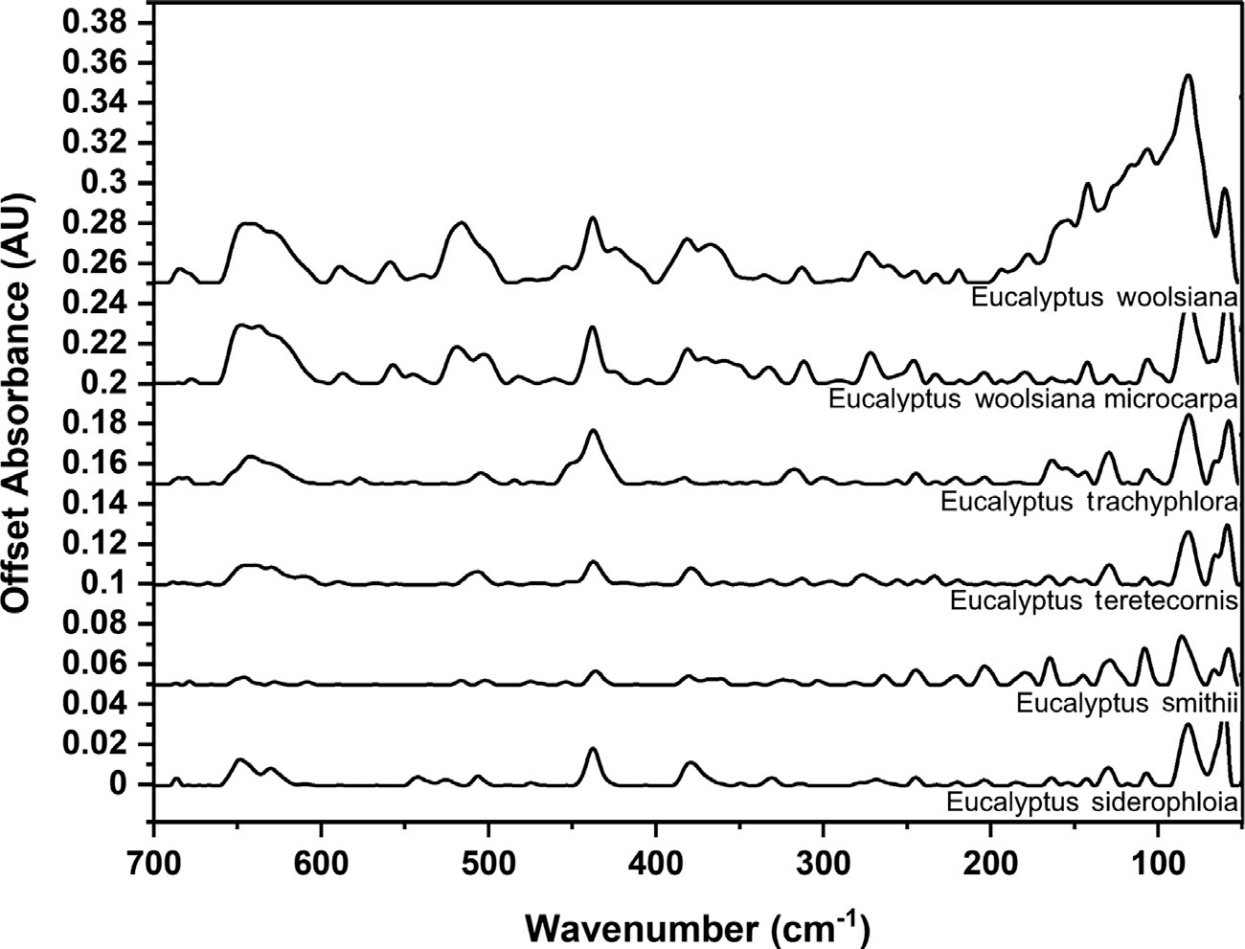
Far-IR spectra for *Eucalyptus* genera (4).

**Fig. 18.**
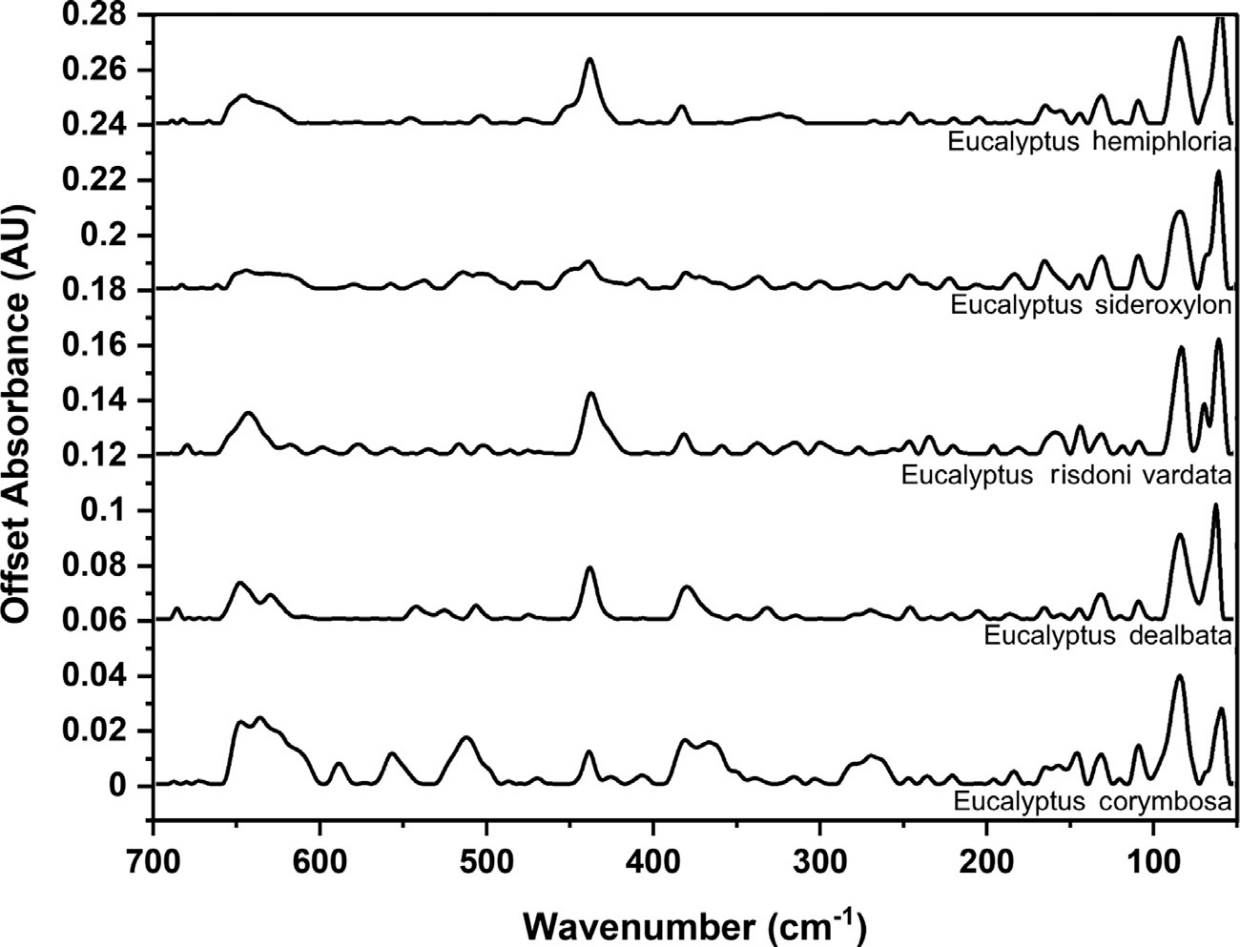
Far-IR spectra for *Eucalyptus* genera (5).

## Ethics Statement

This work abides by all ethical standards and has not included the involvement of human subjects, animal experiments, nor any data collected from social media platforms*.*

## CRediT Author Statement

**Abigail Mann:** methodology, investigation, formal analysis, validation, writing-original draft, writing- review and editing; Dominique **Appadoo:** formal analysis, validation, resources, writing, review and editing; **Claire Lenehan**: conceptualisation, methodology, writing- review and editing; **Rachel Popelka-Filcoff**: conceptualisation, methodology, investigation, writing-original draft, writing- review and editing, supervision, project administration, funding acquisition.

## Declaration of Competing Interest

The authors declare the following financial interests/personal relationships which may be considered as potential competing interests:

Rachel Popelka-Filcoff reports financial support and equipment were provided by The Australian Synchrotron. Rachel Popelka-Filcoff is on the editorial board of Journal of Archaeological Science. The other authors declare that they have no known competing financial interests or personal relationships that could have appeared to influence the work reported in this paper.

## Data Availability

Figshare private linkMann et al. Plant Exudates Mid-IR and Far-IR Data and Analysis (Original data).
